# Case Report: Lack of Response to Givosiran in a Case of ALAD Porphyria

**DOI:** 10.3389/fgene.2022.867856

**Published:** 2022-08-04

**Authors:** Erica Graff, Karl E. Anderson, Cynthia Levy

**Affiliations:** ^1^ Division of Hospital Medicine, University of Miami Miller School of Medicine, Miami, FL, United States; ^2^ Galveston Porphyria Laboratory and Center, Department of Internal Medicine, Division of Gastroenterology and Hepatology, University of Texas Medical Branch, Galveston, TX, United States; ^3^ Division of Digestive Health and Liver Diseases, University of Miami Miller School of Medicine, Miami, FL, United States; ^4^ Schiff Center for Liver Diseases, University of Miami, Miami, FL, United States

**Keywords:** 5-aminolevulinic acid dehydratase, 5-aminolevulinic acid dehydratase deficiency porphyria, 5-aminolevulinic acid synthase, acute porphyria, givosiran, treatment

## Abstract

**Introduction:** 5-Aminolevulinic acid dehydratase (ALAD) porphyria (ADP) is an autosomal recessive disease characterized by a profound deficiency in ALAD, the second enzyme in the heme biosynthetic pathway, and acute neurovisceral attacks with abdominal pain and peripheral neuropathy. Hemin infusions are often effective in treating and preventing such attacks. Givosiran was recently approved for prevention of attacks of acute hepatic porphyrias (AHPs), including ADP, but, to our knowledge, has not yet been applied in patients with this ultrarare disease.

**Case Description:** We update the clinical course and report new treatment outcomes of a 32-year-old man with ADP managed for many years with weekly prophylactic hemin infusions. He has developed evidence of iron overload and was more recently found to have compensated cirrhosis. The patient was started on givosiran (Givlaari™, Alnylam), a small interfering RNA (siRNA) therapeutic that is effective in preventing frequently recurring attacks of acute intermittent porphyria (AIP), the most common type of AHP.

**Discussion:** No adverse effects of givosiran on the liver were observed in this patient with cirrhosis during 6 months of treatment with givosiran. The patient has continued to have recurrent attacks, with transient decreases in ALA levels only as related to treatment of his attacks with hemin. Our experience limited to one patient with ADP suggests that givosiran may not be effective in this type of acute porphyria. Since ADP may have an erythropoietic component, treatment with hydroxyurea, which was beneficial in one previous case, is planned.

## Introduction

5-Aminolevulinic acid dehydratase (ALAD) porphyria (ADP, OMIM 612740) is the rarest of the porphyrias, with only eight cases documented worldwide (Akagi et al., 2006; Lahiji et al., 2020). It is an autosomal recessive disease characterized by a profound deficiency of 5-aminolevulinic acid dehydratase (ALAD) in all tissues and increases in ALA in plasma and urine, coproporphyrin III in urine, and zinc protoporphyrin in erythrocytes (Akagi et al., 2006). The same biochemical abnormalities and neurological symptoms are seen with ALAD inhibition by lead poisoning and by succinylacetone in hereditary tyrosinemia type 2. Porphobilinogen (PBG), the product of ALAD, is normal or only slightly elevated in these conditions as well as ADP. In contrast, the other three more common acute porphyrias are autosomal dominant disorders with approximately half-normal activities of other enzymes in the heme biosynthetic pathway and different patterns of excess heme biosynthetic pathway intermediates including particular elevations of PBG. Attacks of these three AHPs are related to induction of hepatic 5-aminolevulinate synthase 1 (ALAS1), and the first and rate limiting enzyme of heme synthesis in the liver, as evidenced directly by measurement of this enzyme activity in liver and more recently by elevation in exosomal ALAS1 mRNA in plasma and urine (Stölzel et al., 2019). Exosomal ALAS1 mRNA was elevated when measured on one occasion in our patient (Lahiji et al., 2020), but this does not exclude a major erythropoietic component in ADP, which is not present in other AHPs in which erythrocyte porphyrins are not substantially elevated (Lahiji et al., 2020).

In addition, seven of the eight reported ADP patients had biallelic *ALAD* mutations resulting in marked ALAD deficiency and onset of symptoms during childhood or adolescence. The other patient was heterozygous for an ALAD mutation, and ADP developed late in life in association with polycythemia vera, a clonal myeloproliferative disorder. All reported cases to date have been males, in contrast with other AHPs, which are most commonly symptomatic in adult females. Gain of function mutation of 5-aminolevulinate synthase 2 (ALAS2), the erythroid specific form of the first enzyme in the heme biosynthetic pathway, which is encoded by a gene on the X chromosome, was previously excluded in our patient as a potential explanation for male predominance (Lahiji et al., 2020).

The clinical, biochemical, and molecular features of our case of ADP, to date the only documented case in the Americas, have been reported previously (Akagi et al., 2006; Lahiji et al., 2020). His course is updated at this time based on new clinical and treatment findings. He has remained responsive to hemin infusions for prevention and treatment of acute attacks, but after several severe attacks developed permanent foot and wrist drop with contractures, and as reported here, iron overload and cirrhosis. Treatment with givosiran (Givlaari™, Alnylam) was initiated as a potentially better option for prevention of acute attacks. This interfering RNA therapeutic was recently approved for prevention of recurrent attacks of AHP, including ADP, but to our knowledge has not been previously applied in the treatment of ADP. The recent observations in this patient may be of value in guiding management of others with this ultrarare porphyria.

## Case Description and Diagnostic Assessment

This 32-year-old male patient was born in the United States to unrelated parents from Colombia and had no family history of porphyria or liver disease. At age 12, he began experiencing acute attacks of abdominal pain with weakness in his extremities. After about seven attacks in 2 years, acute porphyria was diagnosed based initially on elevated urinary ALA and coproporphyrin. Additional evaluation established a diagnosis of ADP due to two ALAD mutations (c.265G > A, p.Glu89Lys and c.394 T > C, p.Cys132Arg), one inherited from each parent (Akagi et al., 2006). Interestingly, Cys132 is likely involved in coordination of a recently discovered iron–sulfur cluster required for full ALAD activity (Liu et al., 2020). At that time other laboratory testing including ferritin, aminotransferases, amylase, lipase, creatinine, and platelets were normal (Akagi et al., 2006).

His recurrent attacks were treated with hemin (Panhematin™, Recordati) followed by prophylactic hemin 3 mg/kg weekly, which decreased attack frequency to approximately one per year. Serum ferritin increased to 659 ng/ml (ref 32–336) by age 21, which suggested iron overload from repeated hemin infusions, although many ferritin levels were measured within a week after hemin infusions. Weekly small volume phlebotomies reduced his serum ferritin levels to normal, but ferritin became elevated again after phlebotomies were reduced to monthly. Therefore, weekly phlebotomies of 50 or 100 cc before hemin infusions were resumed. Between the ages of 23 and 24, the patient had three attacks despite weekly prophylactic infusions, followed by no attacks for 3 years. At age 30, he had four attacks requiring hospitalization and treatment with hemin within 6 months. At age 31, he developed laboratory evidence of liver disease. Findings included the following: hemoglobin 11.9 g/dl (ref 12.6–17.7), white blood cells 6.4 × 10^3^/ul (ref 3.4–10.8), platelets 134 × 10^3^/ul (ref 150–379), aspartate aminotransferase 74 IU/L (ref 0–40), alanine aminotransferase 75 IU/L (ref 0–44), alkaline phosphatase 263 IU/L (ref 39–117), albumin 4.3 g/dl (ref 3.5–5.5), total bilirubin 0.8 mg/dl (ref 0–1.2), ferritin 405 ng/ml (ref 30–400), iron 106 ug/dl (ref 38–169), total iron binding capacity 348 ug/dl (ref 250–450), and creatinine 1.32 mg/dl (ref 0.9–1.3). Serum electrolytes were normal. Testing for other causes of liver disease included the following: ceruloplasmin 34.7 mg/dl (ref 16.0–31.0), alpha-1-antitrypsin 150 mg/dl (ref 95–164), antinuclear antibody and antimitochondrial M2 antibody negative, anti-smooth muscle antibody 14 U (ref 0–19), and tissue transglutaminase IgA <2 U/ml (ref 0–3). He was homozygous for the H63D *HFE* mutation but negative for C282Y and S65C. Liver biopsy showed nodular architecture, focal sinusoidal dilation, but without significant steatosis or ballooning of hepatocytes. Portal tracts were infiltrated with rare plasma cells and pigmented macrophages. Bile ducts were preserved. Trichrome showed focal partial nodules, stage 3. Prussian blue staining showed excess iron mostly in Kupffer cells and 1 + iron in hepatocytes. Ultrasound with elastography showed heterogeneous echogenicity and a coarse hepatic echotexture, with a mean shear wave velocity of 1.83 m/s consistent with F2-3 (stage 2–3 fibrosis). Both ultrasound and abdominal CT scans showed an enlarged nodular liver suggestive of cirrhosis and splenomegaly, measuring 14.6 cm in the greatest dimension. Work-up for other causes of chronic liver disease was negative except for the finding of homozygous *H63D* mutations of the *HFE* (hemochromatosis) gene. Upper endoscopy showed no esophageal varices, imaging showed no ascites, and he had no symptoms of encephalopathy.

**FIGURE 1:**
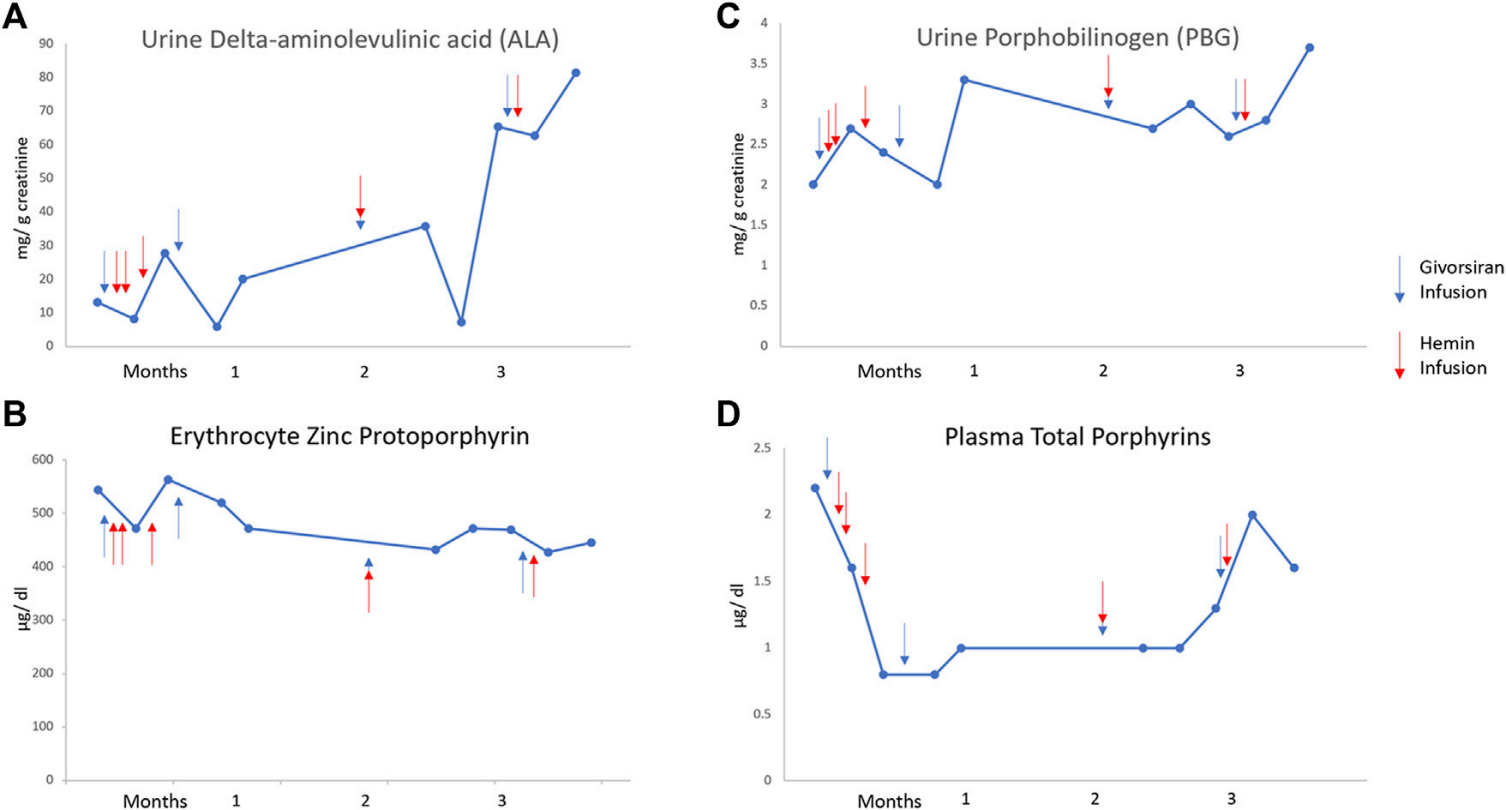
Levels of urinary ALA (top left), urinary porphobilinogen (top right), erythrocyte zinc protoporphyrins (bottom left), and plasma protoporphyrins (bottom right) during 3 months of therapy with givosiran. Hemin and givosiran infusions are denoted by red and blue arrows, respectively. Unfortunately, our patient was hospitalized for a large portion of months 4–6 of givosiran therapy, thus, no subsequent levels are available.

Treatment with givosiran, 2.5 mg/kg subcutaneously monthly was initiated in an effort to reduce ALA levels and prevent attacks and as an alternative to prophylactic hemin. However, after 6 months he has continued to require hemin infusions to treat attacks and small volume phlebotomy for hyperferritinemia. As shown in Figure 1, ALA levels have decreased in the short term after hemin infusions but have not been sustained by givosiran treatment.

## Discussion

This patient with well-documented ADP developed symptoms at age 12 and frequent recurrent attacks continue at age 32. Hemin has been effective for treatment and prevention of attacks until recently. He has remained generally healthy, but has residual wrist and ankle drop because of incomplete recovery of motor neuropathy. At age 32, he now has compensated cirrhosis with evidence of iron overload with elevated serum ferritin levels, which has been treated with small volume phlebotomies before weekly prophylactic hemin infusions. Ferritin levels are difficult to interpret when hemin is administered frequently because hemin can have proinflammatory and other adverse effects (Hopp and Imhof, 2021) causing an acute phase response with an increase in ferritin of uncertain duration.

Givosiran, a 5-aminolevulinate synthase 1 (ALAS1)–directed small interfering RNA (siRNA), which was recently approved for prevention of attacks of AHP, including ADP, was an attractive option for our patient. However, there was no prior experience with givosiran in ADP. There were also safety concerns in this patient recently found to have cirrhosis because in the pivotal phase 3 trial ALT elevation to three times above the upper limit of normal was observed more often with givosiran than with placebo (15%–2%, respectively). These ALT elevations occurred most often three to 5 months after initiating treatment and were independent of baseline ALT levels and often resolved after resumption of drug administration (Balwani et al., 2020). In addition, there was no prior experience with givosiran in patients with cirrhosis since the clinical trials excluded patients with pre-existing hepatic abnormalities. In our patient, ALT and other liver chemistries remained stable throughout treatment with givosiran for 6 months, and without evidence of decompensated cirrhosis, such as hepatic encephalopathy, varices, variceal bleeding, or ascites. This limited experience suggests that givosiran may not pose a substantial risk of further liver damage in patients with pre-existing underlying liver disease. However, further experience in patients with other acute porphyrias and co-existing liver disease is needed.

Patients treated with givosiran in the ENVISION trial, almost all of whom had AIP, had fewer acute attacks and days of hemin use compared to patients on placebo (Balwani et al., 2020). It was anticipated that our patient would respond favorably to givosiran as asialoglycoprotein receptor 1 (ASGR1), the specific receptors for siRNA compounds in parenchymal liver cells, are likely to actually be increased in patients with cirrhosis and without HCC (Witzigmann et al., 2016). However, this treatment does not appear to have changed the disease course in our patient with ADP. He has continued to have frequent acute attacks, all of which have responded to hemin. Moreover, decreases in ALA levels seem related to short term effects of hemin treatment, in contrast to the sustained decreases in ALA and PBG observed with givosiran treatment in patients with AIP (Saberi et al., 2021). As expected, the elevated levels of erythrocyte zinc protoporphyrin have not decreased (Figure 1) because givosiran targets hepatic ALAS1 and not ALAS2, the erythroid specific form of this enzyme.

The patient has also developed compensated cirrhosis with evidence of iron overload. The predominant increase of iron is in Kupffer cells rather than hepatocytes, which suggests that iron deposition is primarily the result of repeated hemin treatment infusions. He was found to have the H63D/H63D *HFE* genotype, which can be a cause of hereditary hemochromatosis, but that condition would be expected to increase iron primarily in hepatocytes (Deugnier and Turlin, 2007). Having developed cirrhosis, he is at increased risk for liver cancer, and surveillance by hepatic imaging is now ongoing. Risk of liver cancer is increased in other acute porphyrias, often in the absence of cirrhosis (Saberi et al., 2021), but to our knowledge has not been observed in ADP.

Evidence that ADP may have a substantial erythropoietic component includes elevation of erythrocyte zinc protoporphyrin, development of the disease in one patient as a complication of a myeloproliferative disorder, and lack of improvement in one case who underwent liver transplantation (Lahiji et al., 2020). This multifactorial etiology of ADP may explain the lack of efficacy of givosiran. Because givosiran has not been effective in our patient, we plan to explore bone marrow suppression with hydroxyurea and transfusions, which was reported to be effective in one patient with ADP (Neeleman et al., 2019).

## Data Availability

The raw data supporting the conclusions of this article will be made available by the authors, without undue reservation.

## Abbreviations

ADP, 5-aminolevulinic acid dehydratase porphyria; AHP, acute hepatic porphyria; ALA, 5-aminolevulinic acid; ALAD, 5-aminolevulinic acid dehydratase; ALAS1, 5-aminolevulinate synthase 1; ALAS2, 5-aminolevulinate synthase 2; ALP, alkaline phosphatase; ALT, alanine aminotransferase; AST, aspartate aminotransferase; HFE, hemochromatosis gene; PBG, porphobilinogen; IU/L, international units per liter; m/s, meters per second; mg/dl, milligrams per deciliter; mg/g creatinine, milligrams per gram of creatinine; mg/kg, milligrams per kilogram; ng/mL, nanograms per milliliter; siRNA, small interfering RNA.
